# Genotypic and phenotypic analysis of *Salmonella enterica* serovar Derby, looking for clues explaining the impairment of egg isolates to cause human disease

**DOI:** 10.3389/fmicb.2024.1357881

**Published:** 2024-06-06

**Authors:** German Matias Traglia, Laura Betancor, Lucia Yim, Andrés Iriarte, José Alejandro Chabalgoity

**Affiliations:** Departamento de Desarrollo Biotecnológico, Instituto de Higiene, Facultad de Medicina, Universidad de la República, Montevideo, Uruguay

**Keywords:** virulence phenotype, acid tolerance, ssaG, mobile elements, outbreaks of salmonellosis, comparative genomics

## Abstract

*Salmonella enterica* serovar Derby causes foodborne disease (FBD) outbreaks worldwide, mainly from contaminated pork but also from chickens. During a major epidemic of FBD in Uruguay due to *S. enteritidis* from poultry, we conducted a large survey of commercially available eggs, where we isolated many *S. enteritidis* strains but surprisingly also a much larger number (ratio 5:1) of *S*. Derby strains. No single case of *S*. Derby infection was detected in that period, suggesting that the *S*. Derby egg strains were impaired for human infection. We sequenced fourteen of these egg isolates, as well as fifteen isolates from pork or human infection that were isolated in Uruguay before and after that period, and all sequenced strains had the same sequence type (ST40). Phylogenomic analysis was conducted using more than 3,500 genomes from the same sequence type (ST), revealing that Uruguayan isolates clustered into four distantly related lineages. Population structure analysis (BAPS) suggested the division of the analyzed genomes into nine different BAPS1 groups, with Uruguayan strains clustering within four of them. All egg isolates clustered together as a monophyletic group and showed differences in gene content with the strains in the other clusters. Differences included variations in the composition of mobile genetic elements, such as plasmids, insertion sequences, transposons, and phages, between egg isolates and human/pork isolates. Egg isolates showed an acid susceptibility phenotype, reduced ability to reach the intestine after oral inoculation of mice, and reduced induction of SPI-2 *ssaG* gene, compared to human isolates from other monophyletic groups. Mice challenge experiments showed that mice infected intraperitoneally with human/pork isolates died between 1–7 days p.i., while all animals infected with the egg strain survived the challenge. Altogether, our results suggest that loss of genes functions, the insertion of phages and the absence of plasmids in egg isolates may explain why these *S*. Derby were not capable of producing human infection despite being at that time, the main serovar recovered from eggs countrywide.

## 1 Introduction

*Salmonella enterica* is a major cause of foodborne disease (FBD) throughout the world. More than 2,600 serovars have been identified, and almost all of them can cause illness in humans and animals, although with marked differences in prevalence and worldwide distribution among them (Issenhuth-Jeanjean et al., 2014). The ability to produce biofilms, the antibiotic resistance profile, and the presence of genes coding for a variety of pathogenicity factors, mainly encoded in the Salmonella Pathogenicity Islands (SPI), contribute greatly to making *Salmonella* a successful foodborne pathogen (Grassl and Finlay, 2008). The SPIs are key determinants of the observed difference in virulence, epidemiology, and pathogenesis between *S. enterica* serovars (Amavisit et al., 2003). Although more than 20 SPIs have been described, only five of them are present in all serovars. There are certainly other conserved and specific mechanisms that play a role in the infective capacity of different serovars, either in the intestine or during the invasion of the host’s tissues (Schmidt and Hensel, 2004).

During infection, *Salmonella* encounters various stress conditions such as high osmotic pressure, low oxygen availability, and the presence of bile salts and antimicrobial peptides that constantly test the fitness of this pathogen. Many different genetic mechanisms involving tolerance to different stress conditions have been reported, some of them independent of the activity of SPI (Foster, 1995; Baik et al., 1996; Wilmes-Riesenberg et al., 1996; Ryan et al., 2015). One such stress condition is the low pH that *Salmonella* encounters during the transit through the stomach, as well as during survival within the *Salmonella*-containing vacuole (SCV) of phagocytic and nonphagocytic cells. Hence, the ability of this species to sense low pH and respond appropriately to such stress is crucial for its survival and pathogenicity (Ryan et al., 2015).

*Salmonella* possesses relevant properties, including virulence factors, resistance to heavy metals, antibiotics, and other traits, which are encoded in plasmids. These replicons carry genes that are dispensable for basic bacterial cell functions but provide increased fitness and are characterized by high evolutionary rates (Rychlik et al., 2006). Therefore, the acquisition of a plasmid may allow the bacteria to respond more quickly to a changing environment.

*Salmonella enterica* subspecies *enterica* serovar Derby (*S.* Derby) is the second most abundant serovar isolated from pork in the European Union and in other parts of the world (EFSA, 2019). This serovar is also usually associated with other sources (Valdezate et al., 2015; European Food Safety Authority and European Centre for Disease Prevention and Control, 2016). Indeed, *S.* Derby was reported as the most prevalent serovar isolated from turkey flocks in the United Kingdom (Hayward et al., 2016). Most recently, it has been included in the top five serovars responsible for human infections in the European Union (European Food Safety Authority, European Centre for Disease Prevention and Control, 2021).

Between 1995 and 2004 there was an epidemic period of outbreaks of salmonellosis due to *S. enterica* serovar Enteritidis in Uruguay, and during 2001–2002, we conducted a countrywide surveillance of more than twelve thousand commercially available eggs from where we isolated several *S. enteritidis* (*n* = 8) that were linked to the isolates causing human infection (Betancor et al., 2010). Strikingly, we also found a much larger number of *S*. Derby (*n* = 39), which, given the lack of *S.* Derby human infections in that period, were presumably non-pathogenic for humans. In this work, we sequenced 14 of these isolates together with 15 other *S*. Derby isolated from humans and pork in other periods in Uruguay and conducted genomic comparisons between them. Our results show that there are marked differences in gene content between egg isolates and isolates from other sources, particularly in the composition of plasmids, insertion sequences, transposon, and phages. Thus, we can hypothesize that these differences are linked to the impaired ability to produce human infection by egg strains.

## 2 Materials and methods

### 2.1 Bacterial isolation

The species identification was performed by Biochemical assays. Serovars were confirmed by agglutination tests with antisera (Difco, Prolab) according to the White–Kauffmann scheme (Grimont and Weill, 2007). The *S.* Derby isolates from human infection and pork were obtained from the National Salmonella Center (Instituto de Higiene, Uruguay). The isolates from eggs were obtained from egg-pools sampled from different food factories (Betancor et al., 2010). A single colony from the original isolates was grown in LB Broth and the bacterial suspensions were stored at −80°C with glycerol 16% for future analysis (Table 1).

**Table 1:** Genome features of Uruguayan *S*. Derby strains sequenced in this study.

Strain	Isolate source	Year of isolate	Length	Contigs (<500 bp)	N50	GC content (%)	CDS	Genes	tRNAs	MLST (STs)	Plasmids type	Biosample/SRA
10/02	Egg	2002	4,771,197	41	316,671	52.07	4,459	4,520	60	40	-	SAMEA2201757 / ERS354160
11/02	Egg	2002	4,788,480	44	307,825	52.07	4,485	4,552	66	40	-	SAMEA2201758 / ERS354161
14/02	Egg	2002	4,781,654	52	215,802	52.06	4,476	4,542	65	40	-	SAMEA2201755 / ERS354158
15/02	Egg	2002	4,789,968	43	213,580	52.07	4,485	4,557	71	40	-	SAMEA2201756 / ERS354159
17/02	Egg	2002	4,790,376	31	473,718	52.07	4,486	4,557	70	40	-	SAMEA2201760 / ERS354163
31/02	Egg	2002	4,790,965	36	307,825	52.07	4,486	4,559	72	40	-	SAMEA2201759 / ERS354162
34/02	Egg	2002	4,789,851	38	419,948	52.07	4,486	4,557	70	40	-	SAMEA2201761 / ERS354164
38/02	Egg	2002	4,774,181	36	419,650	52.08	4,460	4,535	74	40	-	SAMEA2201762 / ERS354165
45/02	Egg	2002	4,785,748	36	356,667	52.07	4,487	4,554	66	40	-	SAMEA2201767 / ERS354170
46/02	Egg	2002	4,785,782	46	290,809	52.05	4,484	4,551	66	40	-	SAMEA2201765 / ERS354168
52/02	Egg	2002	4,788,447	35	421,386	52.07	4,488	4,558	69	40	-	SAMEA2201763 / ERS354166
53/02	Egg	2002	4,789,099	34	375,140	52.07	4,490	4,560	69	40	-	SAMEA2201764 / ERS354167
54/02	Egg	2002	4,787,513	54	264,048	52.06	4,488	4,558	69	40	-	SAMEA2201754 / ERS354157
N11	Egg	2002	4,755,785	37	421,089	52.06	4,443	4,512	68	40	-	SAMEA2201753 / ERS354156
33/09	Human	2009	4,784,756	38	396,101	52.06	4,482	4,550	67	40	-	SAMEA2201752 / ERS354155
16/08	Human	2008	4,806,855	48	347,586	52.04	4,500	4,568	67	40	IncQ2, col.(MGD)	SAMEA2201751 / ERS354154
5/91	Human	1991	4,803,374	43	292,673	52.04	4,532	4,601	68	40	colE1-like	SAMEA2201750 / ERS354153
1/90	Human	1990	4,886,663	46	376,052	52.01	4,627	4,699	71	40	-	SAMEA2201748 / ERS354151
2/85	Human	1985	4,743,330	55	337,231	52.01	4,426	4,493	66	40	colE1-like	SAMEA2201747 / ERS354150
12/84	Human	1984	4,805,127	70	321,778	51.93	4,491	4,556	64	40	IncI1	SAMEA2201742 / ERS354145
17/84	Human	1984	4,875,011	52	340,721	51.93	4,568	4,640	71	40	IncI1, colE1-like	SAMEA2201743 / ERS354146
26/84	Human	1984	4,869,268	66	322,539	51.94	4,565	4,632	66	40	IncI1, colE1-like	SAMEA2201744 / ERS354147
33/84	Human	1984	4,874,232	67	355,793	51.90	4,569	4,637	67	40	IncI1, colE1-like	SAMEA2201745 / ERS354148
3/84	Human	1984	4,811,168	71	402,844	51.91	4,497	4,558	60	40	IncI1, colE1-like	SAMEA2201741 / ERS354144
38/84	Human	1984	4,884,643	63	392,460	51.90	4,580	4,653	72	40	IncI1, colE1-like	SAMEA2201746 / ERS354149
17/92	Pork	1992	4,845,103	55	345,703	51.97	4,563	4,631	67	40	-	SAMEA2201749 / ERS354152
23/83	Pork	1983	4,750,247	55	374,933	52.01	4,430	4,500	69	40	IncI1, colE1-like	SAMEA2201738 / ERS354141
25/83	Pork	1983	4,726,710	50	223,584	52.04	4,410	4,478	67	40	colE1-like	SAMEA2201739 / ERS354142
30/83	Pork	1983	4,765,972	62	374,964	52.00	4,442	4,509	66	40	colE1-like	SAMEA2201740 / ERS354143

### 2.2 Genomes sequencing

Genomic DNA from the *S.* Derby isolates was extracted and purified using the DNeasy Blood and Tissue kit (Qiagen). DNA samples were processed according to Illumina’s instructions for generating paired-end libraries. Paired-end libraries were sequenced using the HiSeq 2000 system – Illumina to a minimum of 30X coverage (Table 1). The total number of produced paired-end reads for each strain ranged from 0.76 × 10^6^ to 8.5 × 10^6^, with a read size of 100 bp. Generated reads were trimmed using Trimmomatic (Bolger et al., 2014), with a Phred score threshold of 30 (Q30). Quality control of the genome sequencing was done by FastQC.^1^

### 2.3 Genome assembly and annotation, comparative and functional genomics analyses

Genome assemblies and assembly quality control were done by SPAdes and QUAST software, respectively (Bankevich et al., 2012; Gurevich et al., 2013). The analyzed dataset also comprised 3,591 genomes of *S*. Derby ST40 downloaded from the Enterobase database (from February 2019). Genome annotations were performed using PROKKA v1.14.5 (Seemann, 2014) and verified with BLASTp (Altschul et al., 1990), both using default parameters. The BLASTp search was done using the protein sequence as the query and the non-redundant protein sequence database at GenBank as the target. We compared the Prokka annotation against the best hit and retained matching annotations. Genome assemblies, annotation, coding and protein sequences are available at zenodo.org/records/11217703.

The functional annotation was performed by InterProScan software and EggNogMapper v2.0 (Jones et al., 2014; Cantalapiedra et al., 2021). The SeqSero online tool was used to validate the serovar from the raw sequence reads (Zhang et al., 2015). MLST (Multi-Locus Sequence Typing) was done by MLST software.^2^ Mutation assessment was determined using the breseq pipeline (Deatherage and Barrick, 2014). Pan-genome analysis and the identification of putative orthologous genes were performed by the ROARY package (Page et al., 2015). The GFF files obtained from the genome annotation were used as input in Roary to obtain the core genome alignment. Variable aligned positions were extracted using SNP-SITES (Keane et al., 2016). A maximum likelihood phylogenetic tree was built using RAXML 8.2.9, the ASC parameter for variable sites, and the RELL bootstrap technique for node support assessment (Minh et al., 2013). The substitution model was predicted by JModelTest2 (Liu et al., 2011; Darriba et al., 2012). The tree representation was done by iTOL (Letunic and Bork, 2021).

The comparative soft-core-genome analysis and functional annotation between egg isolates and human/pork isolates were done by BLASTp (Altschul et al., 1990) and EggNogMapper v2.0 (Cantalapiedra et al., 2021) using default parameter. The soft-core genome was defined as the conserved set of genes present in at least 80% of the genomes of each group. From the comparative soft-core genomes between both groups we identified a set of group-specific genes, being to our study the specific group of genes of egg isolates and human/pork isolates. Group-specific genes are those found in at least 80% of the genomes within one group and in less than 10% of the genomes in other groups.

Bayesian Analysis of Population Structure (BAPS) was done to cluster genotypes using the “fastbaps” R package (Tonkin-Hill et al., 2019). In brief, this software implements a phylogeny-independent approach that employs a nested Bayesian clustering method to analyze population stratification, utilizing core-genome sequences as input data. A logistic principal component analysis (PCA) was performed on a Jaccard distance matrix derived from the original absence/presence data of the accessory genome, using the “vegan” and “FactoMineR” packages in R. Genes present in at least 15% and up to 95% of the analyzed genomes were considered accessory genome and were included in the analysis. Local developed scripts and results of PCA are available online in zenodo.org/records/11217703.

The mobile genetic elements were identified using the ISFinder database (Siguier et al., 2006), PHASTER software (Arndt et al., 2016), PlasmidFinder (Carattoli et al., 2014), Plasmid-Spades software (Antipov et al., 2016), and IntegronFinder (Cury et al., 2016). Antimicrobial resistance and virulence genes were determined using ResFinder and Virulence Factor Database (VFDB), respectively. For the identification of all specific genes, we utilized the BLASTp program with the following parameters: Identity >90%, Coverage >90%, and e-value <10^−6^.

This Whole Genome Shotgun project has been deposited at DDBJ/ENA/GenBank under BioProject accession no. PRJEB4649. BioSamble/SRA identifiers are indicated in Table 1.

### 2.4 Acid tolerance response assays (ATR assay)

The acid resistance assay was performed according to Ryan et al. (2015). Briefly, bacteria were grown overnight at 200 rpm and 37°C in LB broth; the overnight cultures were sub-cultured by 1:100 dilution in acid LB broth (pH 3.3), and then the CFU/ml quantification was performed at different times (0, 2 h, 4 h) by serial dilution and plating onto LBA plates.

### 2.5 Egg white survival assay

Bacterial cell counts of overnight cultures of the *S*. Derby strains were determined by serial dilutions on LB agar plates. A total of 100 μL of 10^5^ CFU/mL bacterial suspensions in PBS was added to 900 μL of egg albumen to obtain a final concentration of approximately 10^4^ CFU/mL egg albumen. Inoculated egg albumen suspensions were incubated at 37°C for 24 h, and then the number of surviving bacteria was determined by plating 100 μL of serial dilutions on LB agar plates. Four independent experiments, with non-inoculated controls in each one, were performed. The survival rate was calculated as a ratio of the bacterial cell counts at 24 h (T24) and the initial bacterial cell counts (T0).

### 2.6 Animal infection models

For mouse infection experiments, bacteria were grown overnight (o/n) at 200 r.p.m. and 37°C in LB broth; the o/n cultures were diluted 1:100 in the same medium and sub-cultured for 3 h at 200 rpm. Bacterial loads in cecal contents were assessed in six to eight-week-old female C57BL/6 mice (n = 5). Briefly, the infection was done by intragastric inoculation with 1–2 × 10^9^ CFU/mL (Inoculation volume: 0.2 mL) of *S*. Derby in PBS. 6 or 24 h post-infection, animals were euthanized, and the cecal content was collected, weighed, and homogenized in PBS. Serial dilutions of the homogenates were plated on SS agar to determine the amount of CFU/gram of cecal content and was calculated using the equation:


$$\frac{CFU}{g}=\frac{\left[\left(\frac{NC*FD}{VS}\right)*VR\right]}{GS}$$

Where NC is the number of colonies, FD is the dilution factor, *VS* is the plated volume, VR is the volume of resuspension of cecal content and GS is the weight of the cecal content expressed in grams.

A human isolate of *S*. Derby recovered from blood (16/08 strain) Was used To evaluate The lethal doses In female CD-1 mice model via peritoneal cavity injection (*n* = 10). Mice were infected with three different doses (10E4, 10E5, and 10E6 CFU/ml, inoculation volume: 0.2 Ml), and The survival rate Was recorded over 2 weeks (Supplementary Figure S1). As a control, The *S. typhimurium* virulent strain ATCC14028 (STM14028) Was used. The optimal infection dose for S. Derby Was 1×10^6^ CFU/mL By peritoneal cavity infection; using this dose, The mice died 24 h post-infection. Thus, survival rates were determined for female CD-1 mice infected with five *S*. Derby strains analyzed In this study (n = 10 mice Per strain). Briefly, 1–2 × 10^6^ CFU/ml (inoculation volume: 0.2 Ml) of *S*. Derby or 1–2 × 10^4^ CFU/mL of *S. typhimurium* STM14028 were injected intraperitoneally. Mice were monitored daily, and The survival rate Was recorded for 2 weeks.

### 2.7 qPCR

Strains were grown to late exponential phase (LEP, OD_600_ 1.0) and early stationary phase (ESP, OD_600_ 2.0) and total RNA isolation was done using RNeasy Minikit (Qiagen). Isolated RNA was then treated with DNase (Invitrogen) and reverse-transcribed using MMLV-reverse transcriptase (Invitrogen) using random primers according to the manufacturer’s instructions. Samples containing no reverse transcriptase or template RNA were included as negative controls to ensure that RNA samples were free of DNA contamination. The *icdA* gene was used as an internal control for normalization in relative quantification. The RT-qPCRs were completed using a QuantiTect kit (Qiagen) with a two-step reaction protocol consisting of 40 cycles of 95°C for 15 s and 60°C for 60 s, followed by a dissociation phase for quality control.

The quantification of each sample was run in triplicate, and experiments were repeated with at least three independent sets of samples. The relative quantification of gene expression was performed by using the 2^-ΔΔC^_T_ comparative threshold method (Livak and Schmittgen, 2001). After normalization, the ratio of cDNA in ESP/LEP for each strain was expressed as a fold change (Relative level transcript). We quantified *ssaG* mRNA levels in ESP related to LEP based on data from Kröger et al. (2013), who reported that several SPI-2 genes, including *ssaG*, are expressed in higher absolute levels in ESP, compared to LEP. Thus, Early Stationary Phase was considered an SPI-2 induction condition while Late Exponential Phase was considered a non-SPI-2 induction condition, and the latter was used for calibration.

### 2.8 Statistical analysis

For analysis of differential bacterial amount in caecal contents (CFU/g) and the transcriptional level (level of mRNA), data was expressed as mean and standard deviations (SD). The Mann–Whitney test was used to study the differences between two groups, and the Kruskal-Wallis test of variance with Dunn’s correction was used to study differences between three or more groups. Survival data were plotted as Kaplan–Meier survival curves and analyzed using the log-rank test. A *p*-value equal to or less than 0.05 was considered statistically significant. All statistical analyses were done using the stats package in R (RR Core Team, 2012).

### 2.9 Ethics statement

Experiments with animals were performed according to national guidelines for animal experimentation that meet the International Guiding Principles for Biomedical Research involving animals, and all protocols were approved by the University Ethics Committee.

## 3 Results and discussion

### 3.1 Egg isolates cluster apart as a monophyletic group

Fourteen *S*. Derby strains isolated from eggs in 2002 and 15 isolates from humans and pork isolated between 1983 and 2008 were sequenced (Table 1) and subjected to comparative genome analyses. A first report describing the polyphyletic nature of *S*. Derby among isolates from pork and poultry in different regions of France found three lineages (ST39-ST40, ST71, and ST682) with different SNP profiles, antibiotic resistance genes, and virulence genes (Sévellec et al., 2018). The same authors reported that two of those lineages, mainly pork isolates corresponding to the MLST profiles ST39-ST40 and ST682, were responsible for 94% of human infections (Sévellec et al., 2020). They also found that while the ST40 was responsible for most human cases (71%), the ST71 was the most prevalent in poultry sources. In our study, ST40 was the unique ST isolated in the country, irrespective of the source of isolation. Genomic differences at the intra-ST level, for instance in the diversity of genomic island or antibiotic resistance profiles, were reported in different *Salmonella* serovars and other bacterial species (Feasey et al., 2016; Decano and Downing, 2019; Hamidian et al., 2019; Hawkey et al., 2019; D’Alessandro et al., 2020). Thus, we performed gene content analysis of all Uruguayan *S.* Derby ST40 isolates to investigate if those recovered from eggs presented genetic differences that could affect their capacity to produce human disease.

Gene content analysis showed 4,179 genes conserved among all Uruguayan isolates, while 13 genes were present in at least one of the egg isolates but absent in all other strains (Supplementary Figure S2 and Supplementary Table S1). Among genes present in at least one isolate from human or pork sources, we identified 524 genes absent in all egg-originated isolates, including mobile genetic elements, antibiotic and metal resistance genes, and putative virulence genes. Instead, most group-specific genes identified in egg isolates were hypothetical protein-coding genes (Supplementary Table S1).

**Figure 1:**
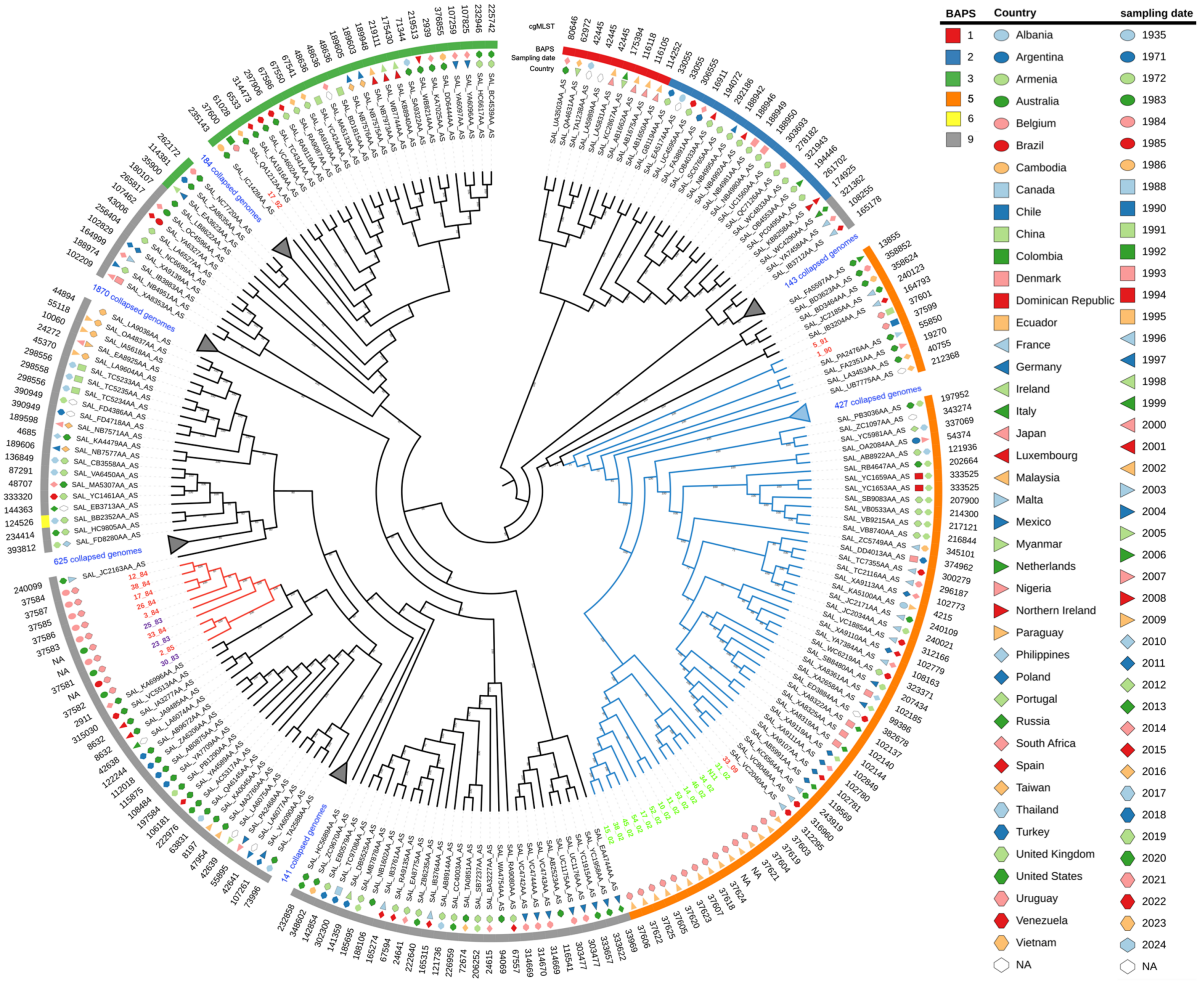
Maximum likelihood phylogenetic tree of the 3,591 *S*. Derby strains analyzed in the present study, with specific lineages collapsed for clarity. The input sequence for phylogenetic analysis comprise 20,126 variable sites identified in 3333 orthologous genes. The RELL bootstrap technique method was used for node support estimation, which is indicated for each branch. The tree representation was done by iTOL. Uruguayan isolates from eggs are written in green, pork isolates in violet, and human isolates in red. Monophyletic groups I and II are indicated by cyan and red branches, respectively. The sampling date, country of isolation, and BAPS1 cluster are provided for each strain. Note that strain 16/08 falls within BAPS cluster four but is not shown because it lies within a collapsed branch. The complete tree figure displaying all nine identified BAPS1 clusters in the dataset is shown in Figure S3, also, a newick tree file is available at zenodo.org/records/11217703.

We performed a core-genome phylogenetic analysis with all 29 Uruguayan strains plus all ST40 *S*. Derby genomes downloaded from the EnteroBase (*N* = 3,591), which comprised isolates from different regions of the world and years of sampling (Supplementary Table S2; Supplementary Figure S3). All fourteen Uruguayan egg isolates were grouped as a monophyletic group with high statistical support. All these egg-isolated strains, along with 472 strains, including the three Uruguayan strains of human origin (5/91, 1/90, and 33/09), grouped together and were named as monophyletic group I (cyan branches in Figure 1). Within this lineage, isolates 5/91 and 1/90 are distantly related to the egg lineage, while 33/90 is its sister lineage. On the other hand, most (10 out of 12) of the other *S*. Derby genomes from Uruguay clustered closely together as a separate group, named monophyletic group II (red branches in Figure 1). The isolates 17/92 and 16/08 fall into different lineages, distant from each other and from both monophyletic groups (Supplementary Figure S3).

We infer population structure by means of BAPS clusters defined at the first level of stratification (BAPS1) (Tonkin-Hill et al., 2019). These genetic clusters, built upon the core genome and encompassing the entire genetic variation in the dataset, objectively define groups of strains sharing similar genetic characteristics, possibly reflecting a common population differentiation process. Altogether, the isolates were divided into nine clusters of BAPS1 (Supplementary Figure S3 and Supplementary Table S2). This is a remarkable result given that all genomes we are working with belong to ST40 and SISTR group B. We observed a substantial but not complete coincidence between the maximum likelihood phylogenetic tree and the generated BAPS clusters (Figure 1, Supplementary Figure S3, and Supplementary Table S2). While monophyletic group I corresponds to BAPS cluster five, monophyletic group II belongs to BAPS cluster nine. The pork isolate 17/92 fall within BAPS cluster three, while human isolate 16/08 fall in BAPS cluster four (Supplementary Figure S3).

We integrated BAPS1 results with a logistic PCA analysis of the accessory genomic content (Gomes-Neto et al., 2023). Thus, the clustering of isolates belonging to the same BAPS1 within the first two principal components reflects a strong co-inheritance between the core genome and the variable genome content. This is the case for most of the BAPS1 clusters generated, for instance clusters three, four, and five (Supplementary Figure S4A), which are mainly separated by the second component of PCA. Cluster nine, which includes all Uruguayan isolates from monophyletic group 2, is widely distributed in the plane generated by the first and second principal components and partially overlaps with the other BAPS1 clusters. This suggests that the accessory genomic content in this group is highly variable, possibly reflecting subpopulation structures not resolved by BAPS1 that could be linked to lineage-specific evolution, involving specific gains and losses of genes. No geographic pattern or year of isolation is associated with the trends defined by the first three components of PCA, which together explain more than 86% of the total variance (PC1 = 44.8%, PC2 = 29.3%, and PC3 = 12.7%). This indicates that the accessory genomic content may be independent of both the year and country of isolation (see Supplementary Figures S4B,C). This could be, at least partially, associated with the widespread distribution of successful ST40 lineages across different times and geographical locations.

The distribution of Uruguayan strains in the plane generated by the first two components of the PCA matched the branch pattern observed in the phylogenetic tree (Supplementary Figure S4D). Consequently, strains from each monophyletic group cluster together and do not overlap with the other monophyletic group. Interestingly, strain 17/92 overlapped with monophyletic group II, while strain 16/08 is distant from all Uruguayan strains. These observations support the idea that both monophyletic groups compared represent distinct subpopulations with independent evolutionary trajectories.

Additionally, notable differences exist in the accessory genome between these groups. However, since there are human isolates in monophyletic group I, these differences may not fully explain the egg-isolated impairment of causing human infection. Antimicrobial resistance (AMR) genes have been searched within Uruguayan strains, resulting in the identification of seven distinct AMR genes present in isolates from both human and pork sources. Conversely, no AMR genes were detected in isolates obtained from eggs (Supplementary Figure S5). The prediction of virulence genes (VGs) was conducted, revealing the presence of 92 VGs in Uruguayan isolates grouped within monophyletic group II, collected from both human and pork sources. Conversely, all but one VG were detected in Uruguayan isolates from monophyletic group I. Notably, the *sspH1* gene, which encodes an effector of the Type 3 Secretion System, emerged as a distinguishing gene which is probably absent in all BAPS1 cluster five (Supplementary Figure S5). SspH1 is a Type 3 secretion system effector encoded in the Gifsy-3 prophage and variably present among isolates from different *Salmonella* serovars (Figueroa-Bossi et al., 2001; Herod et al., 2022). It has been demonstrated that SspH1 contributes to persistence during systemic infection in mouse models (Kidwai et al., 2013) and mediates the degradation of the mammalian protein kinase PKN1 during infection of macrophages *in vitro* (Herod et al., 2022). Therefore, the absence of SspH1 may potentially contribute to the differential infectivity observed among Uruguayan isolates within monophyletic groups. Functional annotation and clustering of the group-specific genes, allowed us to identify 92 genes enriched in the egg isolates and 31 genes overrepresented in isolates from human or pork sources (Supplementary Table S3), which exhibit distinctive patterns between the groups. Notably, many of these genes are hypothetical proteins and are associated with phage-related sequences. This result suggests that the insertion of phages within genomes may also contribute to the differential phenotypes observed.

There is a potential bias introduced by the comparative genome analysis of isolates collected over vastly different time frames. Strains of egg origin were collected during a short period of time, while human/pork isolates were collected over a period spanning more than 20 years. To mitigate this bias in future studies, it may be necessary to sequence and analyze the genomes of *S*. Derby egg-originated strains isolated around a similar time frame from lineages that did not cause human infections. Such a comparison could provide stronger support for the genetic changes proposed here that contribute to the inability of egg isolates to cause human disease.

### 3.2 Egg isolates have an acid susceptibility phenotype

**Figure 2:**
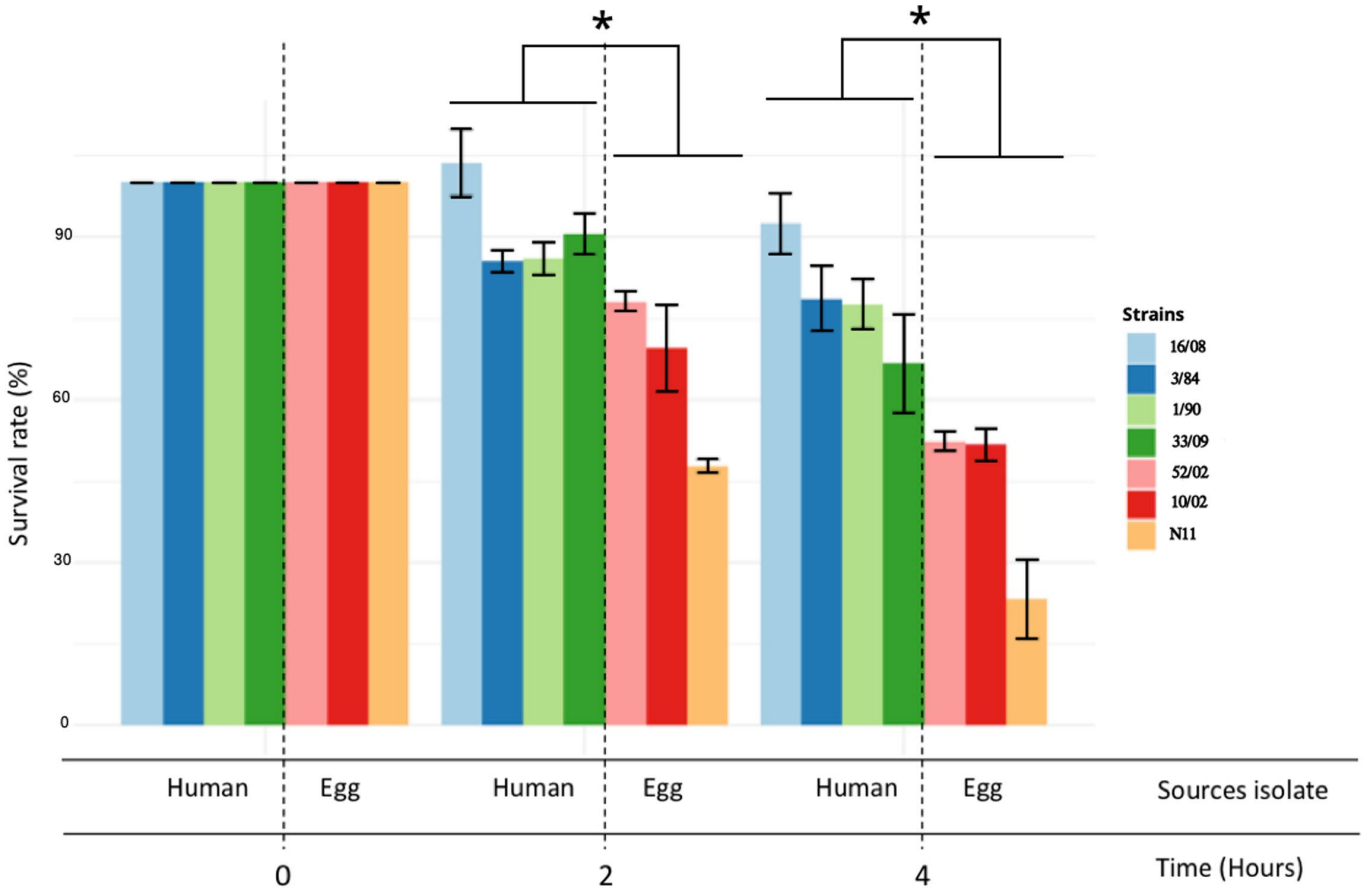
Acid tolerance response and comparison between egg and human isolates. The acid tolerance response was represented as a percent of survival rate (Kruskal-Wallis test and Dunns post-test, *p < 0.05). Survival rate was measured at 2 and 4 h. The standard deviation is indicated as error bars.

**Figure 3:**
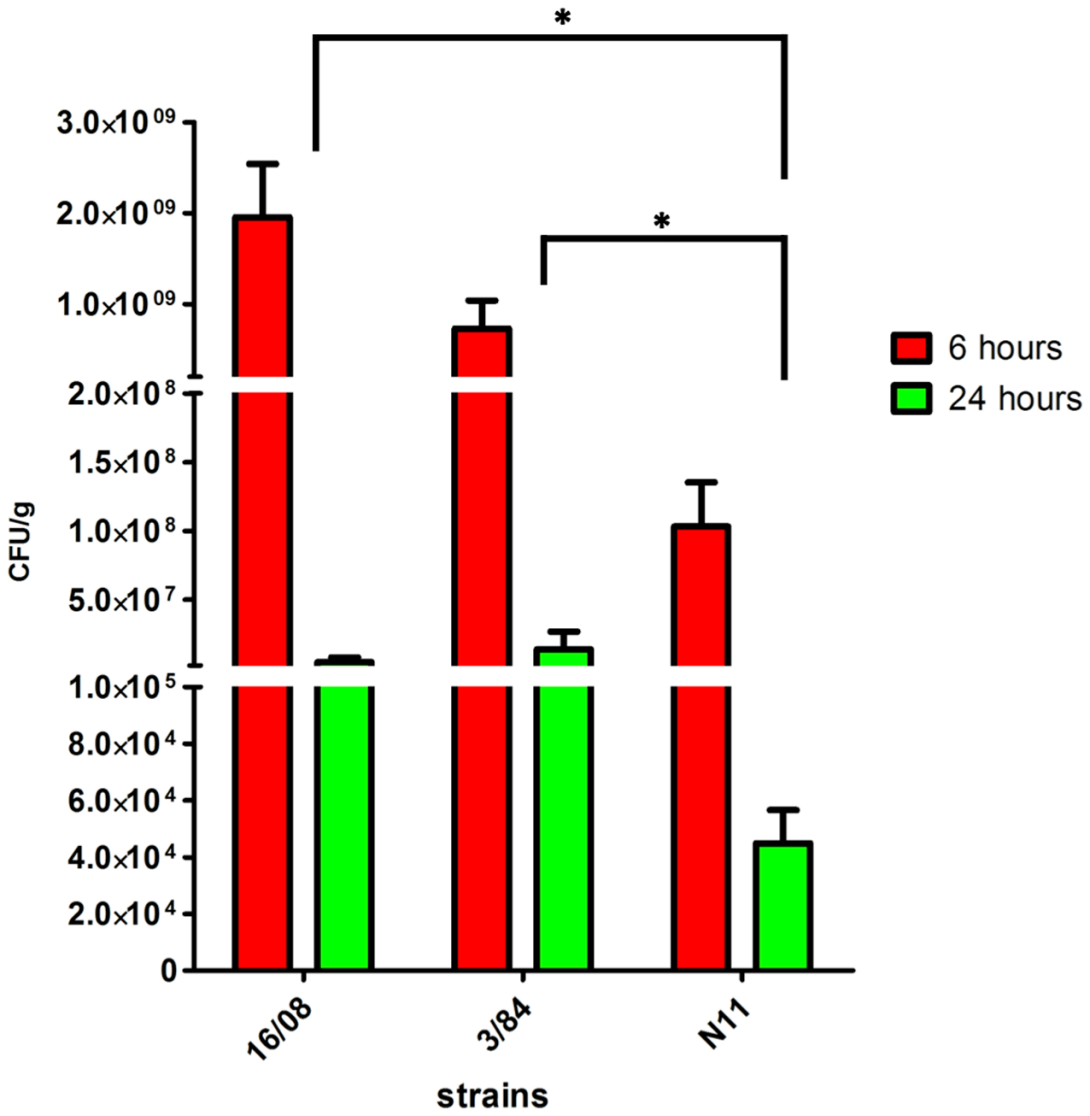
Bacterial count in the caecal content of C57BL/6 mice infected with *S*. Derby (n = 5 mice per group). The bacterial count was represented as CFU by grams of caecal content (Kruskal-Wallis test and Dunns post-test, *p < 0.05). One representative strain from egg-isolates (N11), one strain from human origin from monophyletic group II (3/84) and the non-related isolate 16/08 were included in the analysis. Performed bacterial quantification in the cecal content was done after 6 and 24 h. The standard deviation is indicated as error bars.

The acid tolerance response in *S. enterica* plays an important role in its capacity to survive passage through the stomach (Baik et al., 1996; Viala et al., 2011). To be an effective foodborne pathogen, *S*. Derby needs to resist acid stress in the stomach during the infection process before it can colonize the ileum and cecum. Thus, we selected three-egg isolates (N11, 52/02, and 10/02) and four human isolates (3/84, 16/08, 33/09, and 1/90 strains) and tested them for acid tolerance by incubation at pH 3.3 (LB broth, pH 3.3). We found significant differences in the capacity to survive in an acid medium, with egg isolates showing consistently less acid tolerance than human isolates (Kruskal-Wallis, *p* < 0.05) (Figure 2). Then, we infected C57Bl/6 mice by the oral route with one representative strain from egg isolates (N11), one strain of human origin from monophyletic group II (3/84), and the non-related human isolate 16/08 and performed bacterial quantification in the cecal content after 6 and 24 h. We observed that the egg isolate was recovered in significantly fewer numbers than the other two strains (Figure 3). Based on this, we hypothesize that egg isolates are poorly adapted to survive in acid conditions, and this can explain, at least in part, why these isolates were unable to infect humans.

Gu et al. (2021) reported a list of 35 genes involved in acid stress tolerance in *S*. Derby using Tn-seq and phenotype assays. We analyzed the variability of 32 of these genes, which could be mapped in Uruguayan strains’ genomes. We found no conclusive differences between egg and human/pork isolates, although more variability was observed in monophyletic group I when analyzing sequences at both the protein and nucleotide levels (Supplementary Table S4).

### 3.3 Egg isolates show a reduced expression of SPI-2 *ssaG* gene

**Figure 4:**
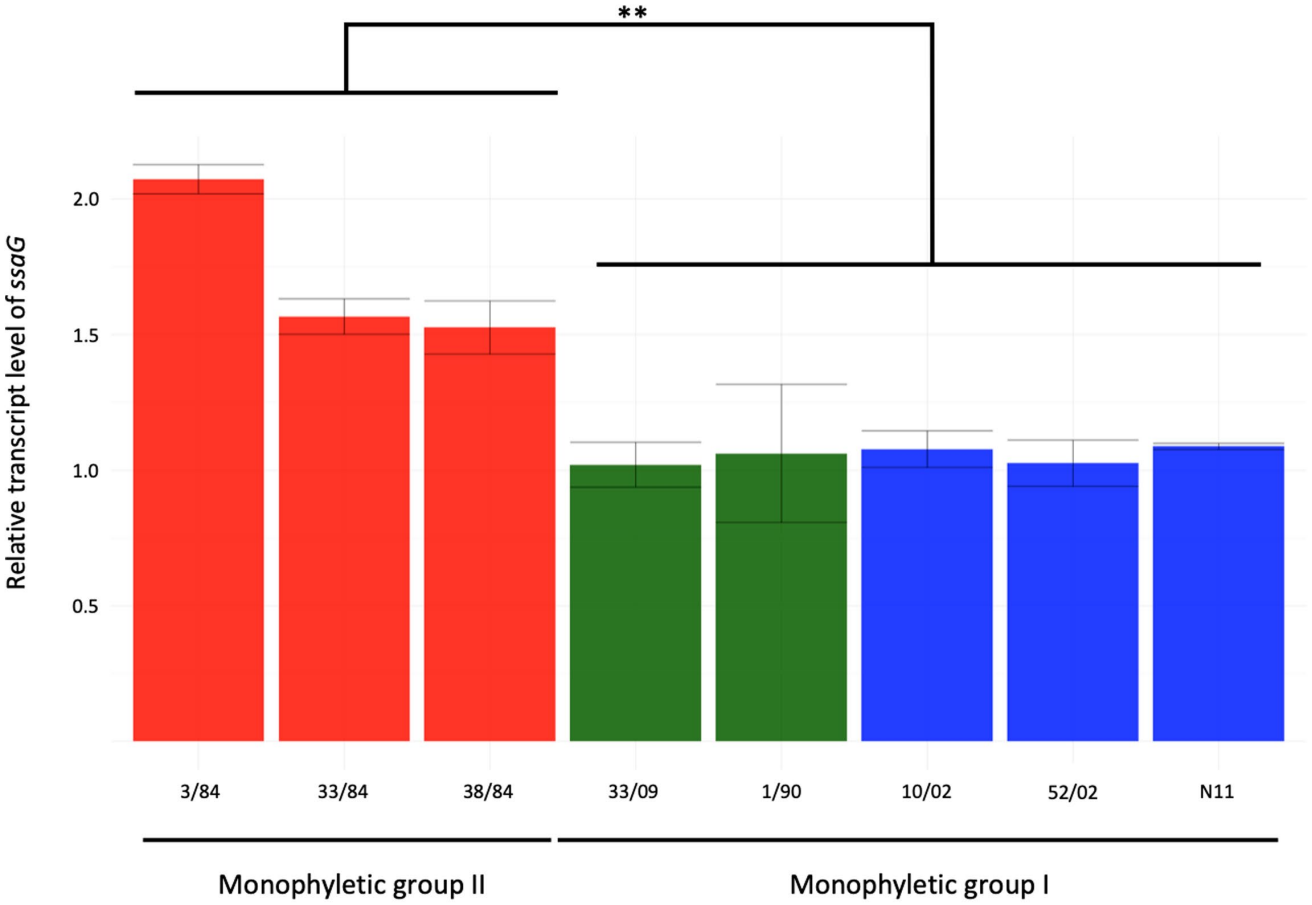
Relative expression of *ssaG* gene in the inductive condition of ESP (OD_600_ = 2) of eight strains of *S*. Derby. The isolates analyzed included five from monophyletic group I (three from eggs and two from humans in blue and green bars, respectively) and three isolates from monophyletic group II (red bars). Kruskal-Wallis and Dunns post-analysis were performed (^**^p < 0.01, *n* = 3). The standard deviation is indicated as error bars.

*Salmonella* pathogenicity island 2 (SPI-2) encodes a type III secretion system (T3SS-2) needed for bacterial survival and replication in the host cells’ intracellular environment (Fulde et al., 2021). To evaluate SPI-2 activity as a main *Salmonella* virulence factor, we assessed the expression of the *ssaG* gene, coding for a TTSS-2 needle protein, in an SPI-2 induction condition related to a condition where it is not expressed (Kröger et al., 2013). We included five strains from monophyletic group I (three egg- and two human-isolated strains) and three from monophyletic group II (all three isolated from humans). We observed between 1.5- and 2-fold increase in *ssaG* mRNA levels in the SPI-2 induction condition related to calibrator condition in strains from the monophyletic group II (3/84, 33/84, 38/84), while strains from monophyletic group I (N11, 10/02, 52/02, 1/90, 33/09) showed no increase (Figure 4).

**Figure 5:**
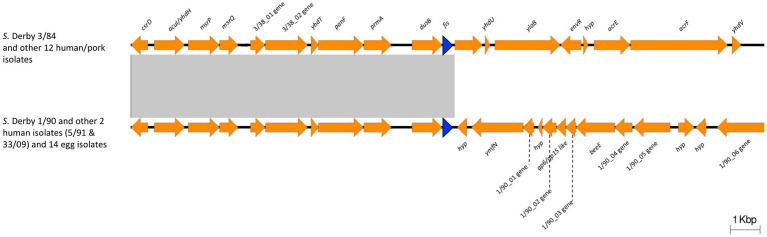
Comparison of the genomic context of *fis* gene between Monophyletic group I and II. The blue arrow indicates the *fis* gene. 3/38_01 gene: Biotin carboxylase of acetyl-CoA carboxylase; 3/38_02 gene: Biotin carboxyl carrier protein of acetyl-CoA carboxylase; 1/90_01 gene: phage terminase small subunit gene; 1/90_02 gene: HNH endonuclease gene; 1/90_03 gene: phage head closure protein; 1/90_04 gene: HK97 family phage prohead protease; 1/90_05 gene: phage major capsid protein; 1/90_06 gene: phage tail tape measure protein. Note the insertion of a phage-originated sequence downstream the *fis* gene in Monophyletic group I. The conserved region is indicated with a grey box.

Expression of *ssaG* is regulated by genetic context and various stress conditions. Lim et al. (2006) reported that *ssaG* expression is regulated by Fis transcriptional factor, which binds to Fis-site promoter sequences upstream to *ssaG*. Despite the observed differences in *ssaG* expression, we analyzed the Fis-site sequences of *ssaG* among various isolate groups and found no genetic differences between them (Supplementary Figure S6). Moreover, we assessed for differences in Fis protein, and the alignment of the Fis amino acid sequences showed 100% identity among all the *S*. Derby genomes (data not shown). However, when we analyzed and compared the genetic context of the *fis* gene among the *S*. Derby genomes, we identified different genetic rearrangements between isolates from monophyletic groups I and II (Figure 5). All Uruguayan strains from the monophyletic group I presented the insertion of a phage-originated sequence downstream of the *fis* gene that could modify its expression (Osmundson et al., 2012; Carey et al., 2019; Nilsson et al., 2022). This modification could occur through the acquisition of a regulatory sequence, replacing the natural promoter, or through a gene that alters the expression of closely located genes. The deregulation of *fis* could generate some modification in the expression of *ssaG*. However, we analyzed *fis* expression by RTqPCR and observed no differences in the transcriptional level among strains analyzed from both monophyletic groups (Supplementary Figure S7). We speculate that the differences in the regulation of *ssaG* expression between the two groups of isolates could be explained by different expressions of other transcriptional factors (SsrAB, PhoPQ), an unknown non-coding RNA or a regulatory gene acquired in the phage downstream of *fis*. The differential regulation of *ssaG* between the two monophyletic groups requires further studies that will be conducted.

### 3.4 Mobile genetic elements differ markedly between Uruguayan isolates from both monophyletic groups

We evaluated the presence of mobile genetic elements, such as insertion sequences, putative phages, integrons, genomic islands, and plasmids. Our analysis revealed considerable differences between Uruguayan strains from both monophyletic groups. Twelve different ISs were identified in the sequenced genomes. Notably, ISEc33, ISSen7, ISRor2, IS630, IS1351, ISSsu9, ISCep1, and IS200F are conserved among all analyzed Uruguayan isolates. All isolates in monophyletic group II have ISErsp1, which is absent in isolates from monophyletic group I. On the other hand, the Tn1331 transposon is also absent in monophyletic group I and is only identified in four out of the seven human isolates from monophyletic group II. Interestingly, the Tn1331 transposon contains the antibiotic resistance genes: *bla_OXA-9_*, *aadA1*, *aac(6′)-Ib,* and *bla_TEM-1_*. Tn1331 was previously reported in different serovars of *Salmonella* in Argentina, but none of them was Derby (Orman et al., 2002).

We identified nineteen putative prophages among *S*. Derby genomes (Supplementary Figure S8 and Supplementary Table S5). One prophage, a BcepMu-like phage, was found in all isolates. Strikingly, we observed differences in the prophage content between both monophyletic groups. The isolates from monophyletic group I contain the SFV-like phage, while the monophyletic group II contains the SAL3-like phage that was reported as a prophage with wide distribution in *Salmonella* (Mottawea et al., 2018). Besides, the prophage composition in monophyletic group II is highly variable.

We found that none of the egg isolates harbor plasmids, while two different types of plasmids were identified in isolates from monophyletic group II: one large conjugative plasmid (IncI) and one small plasmid (ColE1-like) (Table 1; Supplementary Figure S5).

The IncI1 plasmid contains different antibiotic resistance genes and several other accessory genes that could be related to the ability of *S*. Derby to survive and persist in the host. Bleicher et al. (2013) reported the co-occurrence of the IncI1 large plasmid, which contains genes associated with conjugation as well as with antibiotic resistance, and a ColE1-like plasmid in *S*. Derby isolates recovered from pork in Germany. Also, Oladeinde et al. (2018) reported the presence of ColE1 plasmids as triggers of biological fitness and virulence capacity of *S*. Heildelberg. Thus, we hypothesize that the absence of plasmids in *S*. Derby isolates from eggs could be related to their diminished ability to cause human infection. In other words, the loss of these plasmids, which typically carry genes associated with pathogenicity, antibiotic resistance, and metabolic capabilities, may be linked to the bacteria’s reduced ability to acquire new virulence genes, survive, and proliferate within the human body.

Also, we identified and characterized the SPI genomic islands using blast against the main SPIs sequences described in the literature (Amavisit et al., 2003; Hayward et al., 2013). We found 11 out of 23 SPIs identified in *S. enterica* (SPI-1, −2, −3, −4, −5, −6, −9, −11, −16, −18, and − 23). The different SPIs detected in *S*. Derby present the same genetic structure in all analyzed isolates. Thus, we conclude that the virulence phenotype differences were not associated with genetic rearrangement in SPIs.

The absence of plasmids, as well as the particular ISs content and phage profile observed in egg isolates, could be related to an adaptation process to the egg environment. Thus, strains isolated from egg sources may have some tropism for these matrices and act as commensal bacteria in laying hens. Here, we evaluated the isolates in their ability to survive in egg white and did not detect significant differences between different groups of isolates (Supplementary Figure S9). This result indicates that the different profiles of mobile genetic elements are unrelated to the adaptation to the egg environment. Further analyses are needed to elucidate whether the observed lineage-specific profile of mobile genetic elements responds to any adaptation process.

### 3.5 *S*. Derby isolates from the monophyletic group I present an attenuated behavior in a mouse infection model

**Figure 6:**
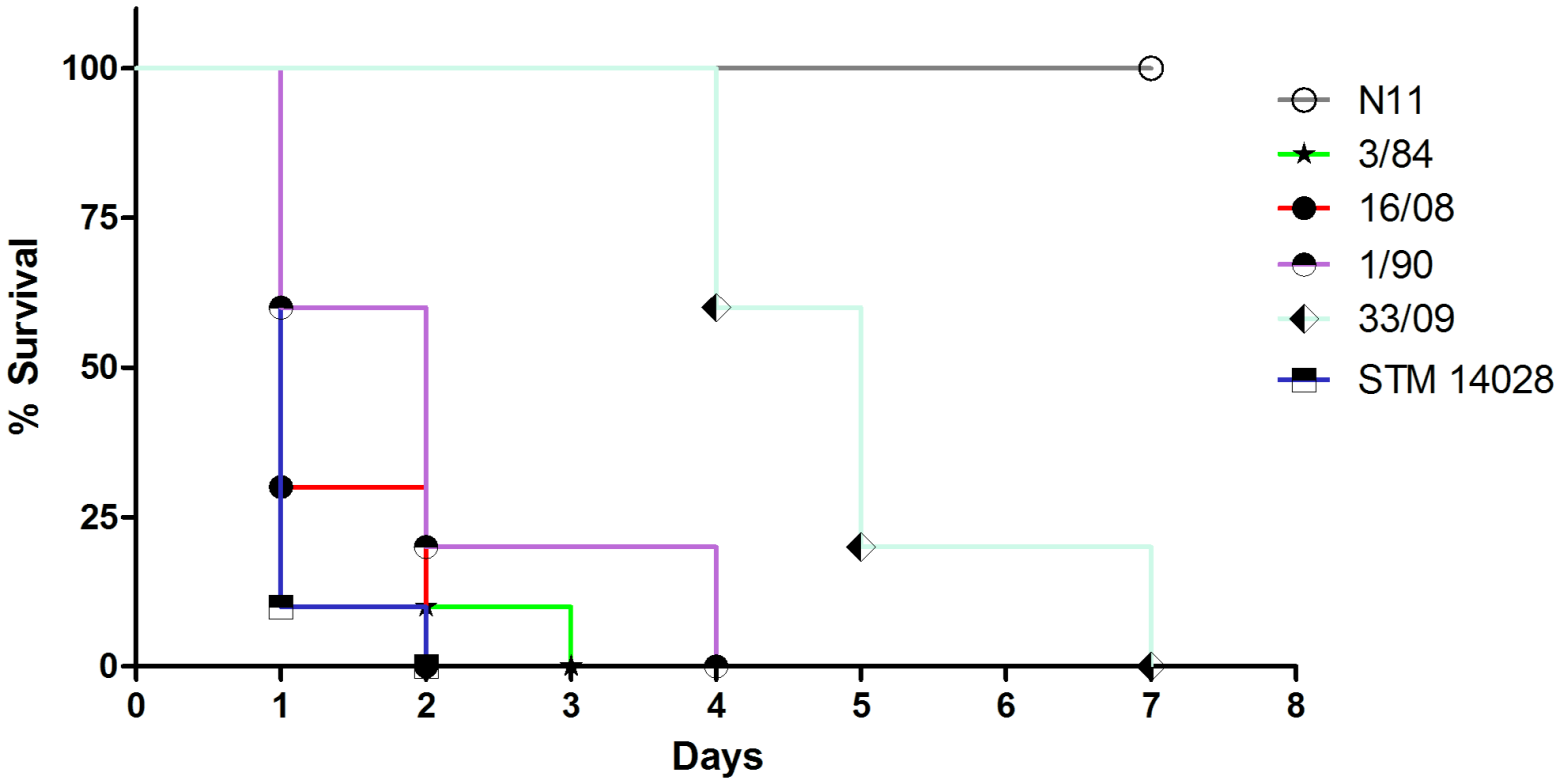
Survival curve of CD-1 mice infected by i.p. route with different strains. 10 animals were infected with 0,2 mL of *S*. Derby suspension 1-2×10^6^ CFU/ml or *S. typhimurium* 1×10^4^ CFU/ml. The analyzed isolates encompass three representatives from monophyletic group I (N11 isolated from eggs; 33/09 and 1/90 from human sources), one human isolate from monophyletic group II (3/84), and one human isolate from Cluster A (16/08). *S. typhimurium* STM 14028 serves as reference strain and positive control of death.

To evaluate the pathogenicity of the different *S*. Derby isolates, we determined the survival rate of mice infected intraperitoneally with isolates from different sources and phylogenetic groups. We evaluated one egg isolate (N11 strain) and 2 human isolates from monophyletic group I (33/09 and 1/90), one isolate from the monophyletic group II (3/84), and the 16/08 isolate belonging to the distant phylogenetic cluster A (Figure 1). Isolates 3/84 and 16/08 induced significant mortality, whereas N11 demonstrated an inability to kill mice. Conversely, both human isolates from monophyletic group I (1/90 and 33/09) exhibited an intermediate level of mortality (Figure 6).

## 4 Conclusion

During an epidemic of foodborne disease (FBD) caused by eggs contaminated with *S. enteritidis* in Uruguay, extensive egg contamination with *S*. Derby was also observed, yet it did not lead to detected human infections. Comparative genomic analysis revealed that all analyzed circulating *S*. Derby during that period belong to ST40. Noteworthy, *S*. Derby strains causing human infections, isolated at other different periods in the country, are not closely related to those isolated instead, they form two clearly separated monophyletic groups with marked differences in gene content and phenotypic traits between them. Genetic differences include plasmid content, phages, and other mobile elements. Phenotypic studies indicated that strains isolated from eggs during the epidemics exhibit lower acid tolerance and induction of expression of *ssaG*, as well as reduced virulence in mice, providing insights into the observed epidemiological behavior. We conclude that the loss of key virulence genes, the insertion of phages and the absence of plasmids in egg isolates may explain the incapacity of these *S*. Derby strains to cause human infection despite being, at the time, the predominant serovar in eggs nationwide.

## Data availability statement

The datasets presented in this study can be found in online repositories. The names of the repository/repositories and accession number(s) can be found in the article/Supplementary material.

## Ethics statement

The animal study was approved by University Ethics Committee (Comité de ética de la Facultad de Medicina, Universidad de la República, Uruguay). The study was conducted in accordance with the local legislation and institutional requirements.

## Author contributions

GT: Data curation, Formal analysis, Investigation, Methodology, Software, Validation, Visualization, Writing – original draft, Writing – review & editing, Conceptualization. LB: Conceptualization, Investigation, Writing – review & editing. LY: Conceptualization, Investigation, Writing – review & editing. AI: Conceptualization, Methodology, Resources, Supervision, Writing – review & editing. JC: Conceptualization, Funding acquisition, Investigation, Project administration, Resources, Supervision, Writing – review & editing, Methodology.

## Footnotes

^1^ www.bioinformatics.babraham.ac.uk/projects/fastqc/

^2^ https://github.com/tseemann/mlst
