# Supplementary material for: Genotypic and phenotypic analysis of Salmonella enterica serovar Derby, looking for clues explaining the impairment of egg isolates to cause human disease
Source: Front Microbiol. 2024 Jun 6;15:1357881. doi: 10.3389/fmicb.2024.1357881 (PMC11186997; doi:10.3389/fmicb.2024.1357881)
Supplement: Supplementary file 6 [file Image_1.PDF]

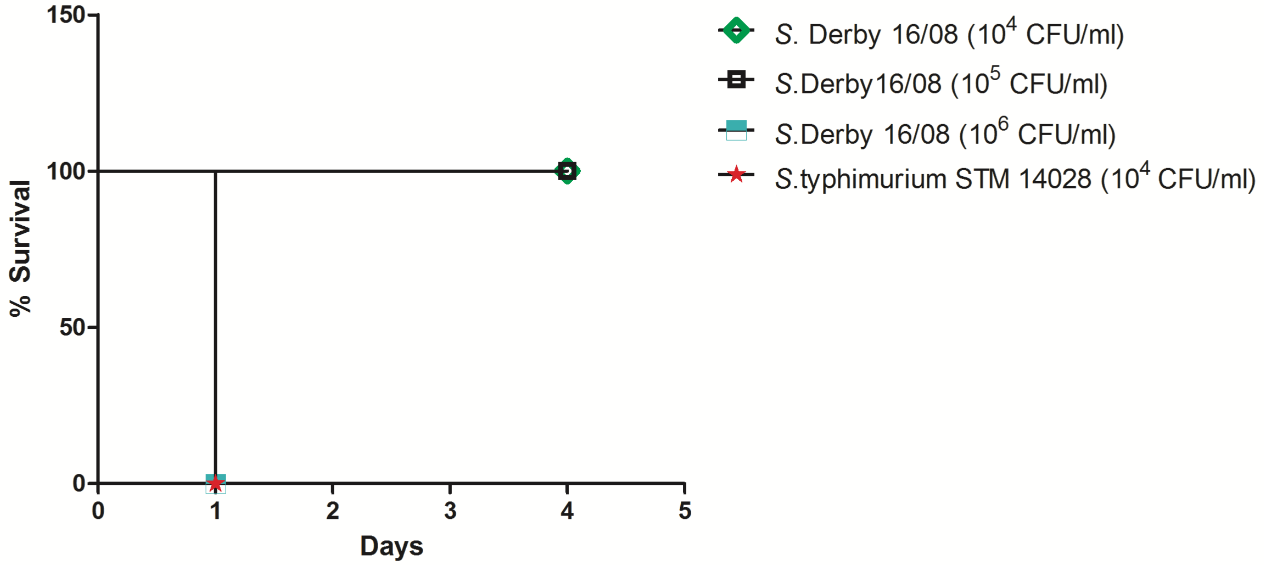

Figure S1. Survival curve of CD-1 mice infected by i.p. route with *S*. Derby 16/08 strain or *S*. Typhimurium 14028. 10 animal per group were infected with 0,2 mL of bacterial suspension. Infection doses are denoted in the survival curves.
