# Supplementary figures and images for: Genotypic and phenotypic analysis of Salmonella enterica serovar Derby, looking for clues explaining the impairment of egg isolates to cause human disease
Source: Front Microbiol. 2024 Jun 6;15:1357881. doi: 10.3389/fmicb.2024.1357881 (PMC11186997; doi:10.3389/fmicb.2024.1357881)

Isolate Source

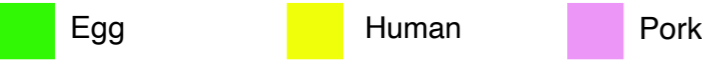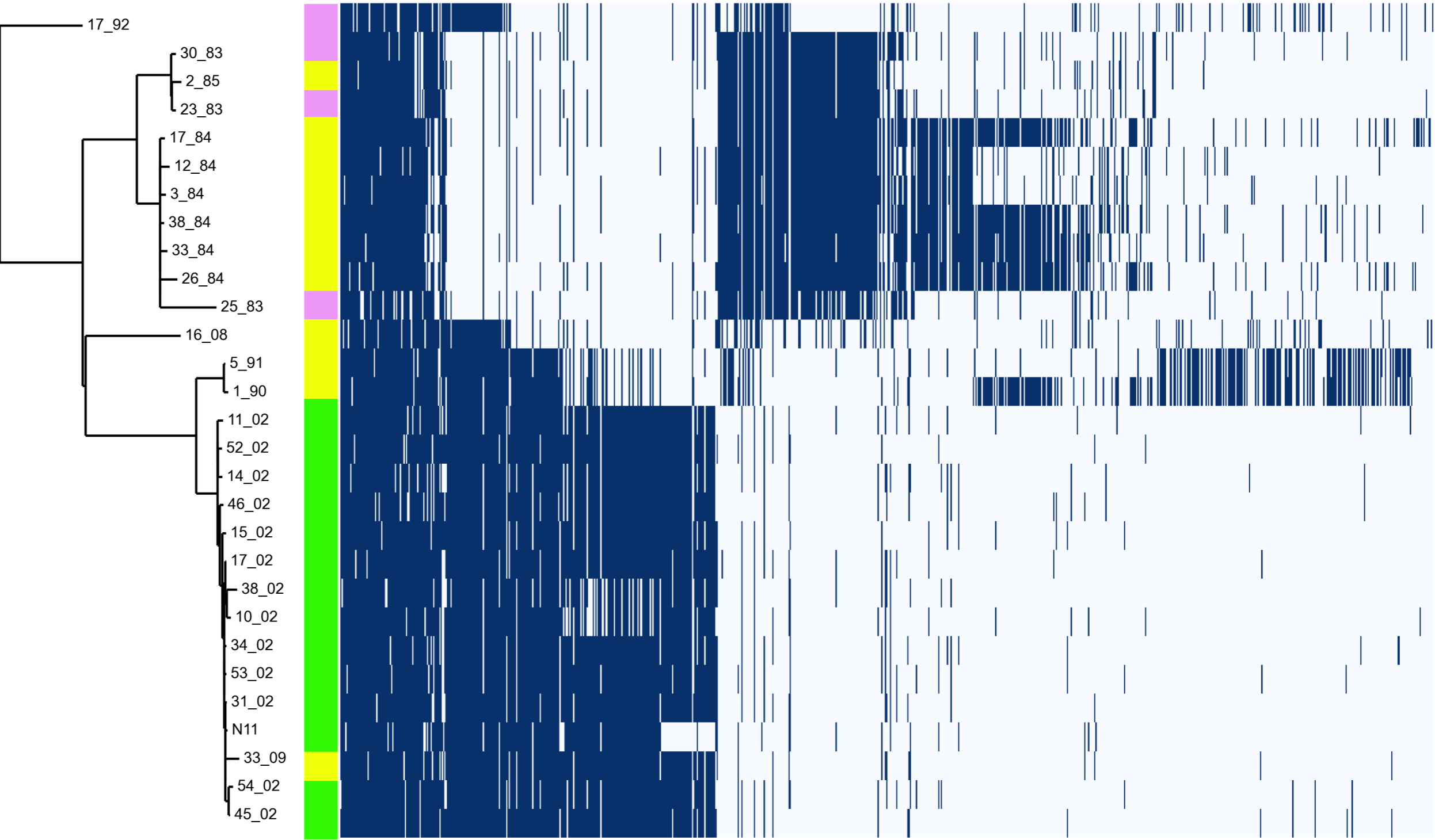

Supplement: Supplementary file 7 [file Image_2.PDF]
