# Supplementary material for: Genotypic and phenotypic analysis of Salmonella enterica serovar Derby, looking for clues explaining the impairment of egg isolates to cause human disease
Source: Front Microbiol. 2024 Jun 6;15:1357881. doi: 10.3389/fmicb.2024.1357881 (PMC11186997; doi:10.3389/fmicb.2024.1357881)
Supplement: Supplementary file 8 [file Image_3.PDF]

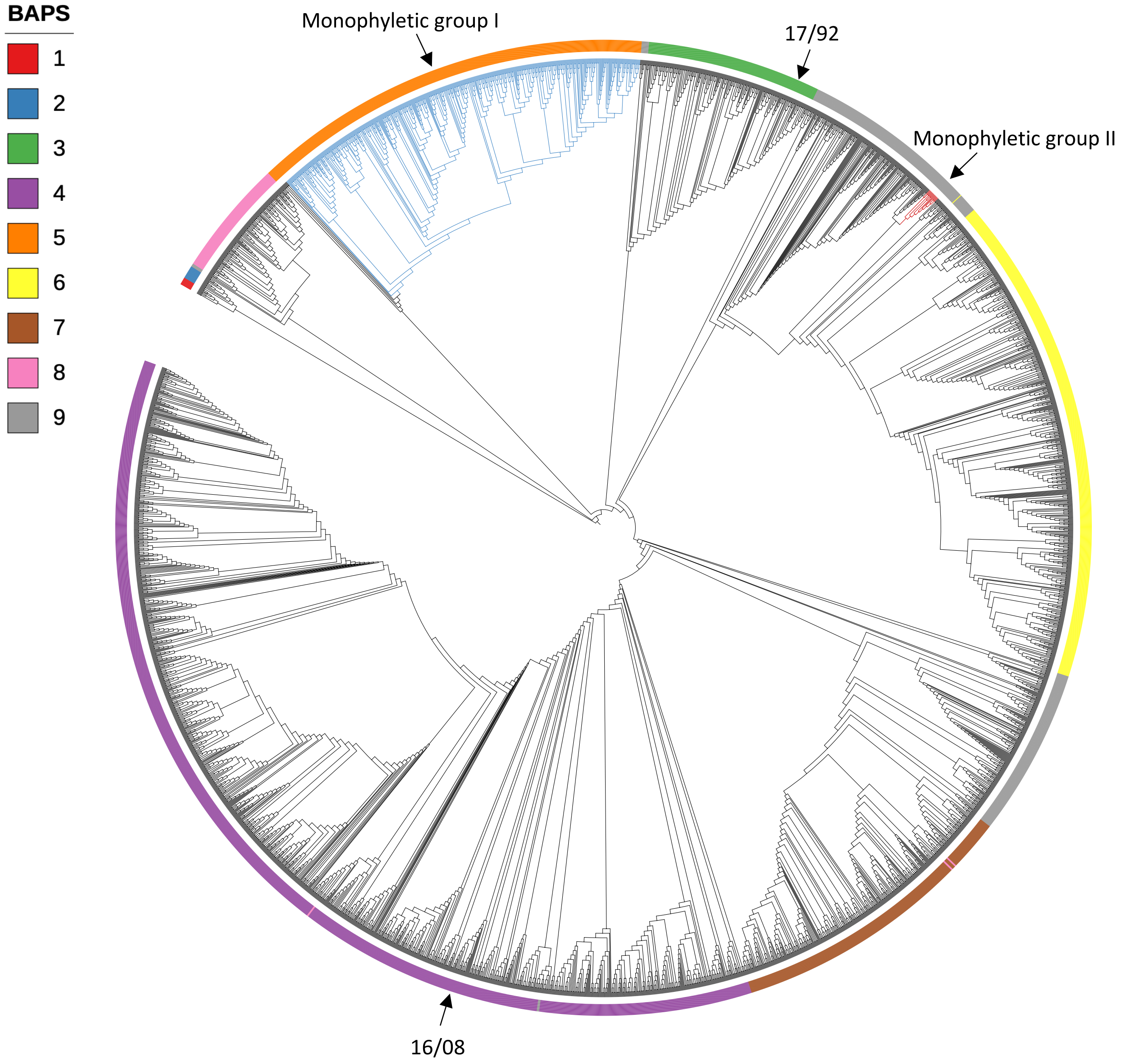

Figure S3. Maximum likelihood phylogenetic tree of 3591 S. Derby strains analyzed in this study. The input sequences for phylogenetic analysis consisted of 20,126 variable sites identified across 3333 orthologous genes. The tree was visualized using iTOL. Monophyletic groups I and II are highlighted by cyan and red branches, respectively. The position of Uruguayan strains 16/08 and 17/92 are indicated. BAPS1 clusters are labeled for each strain. Additional strain information can be found in Table S2. The complete tree file (in Newick format), including estimated node support and distances, is available in the Zenodo repository: zenodo.org/records/11217703.
