# Supplementary material for: Genotypic and phenotypic analysis of Salmonella enterica serovar Derby, looking for clues explaining the impairment of egg isolates to cause human disease
Source: Front Microbiol. 2024 Jun 6;15:1357881. doi: 10.3389/fmicb.2024.1357881 (PMC11186997; doi:10.3389/fmicb.2024.1357881)
Supplement: Supplementary file 9 [file Image_4.PDF]

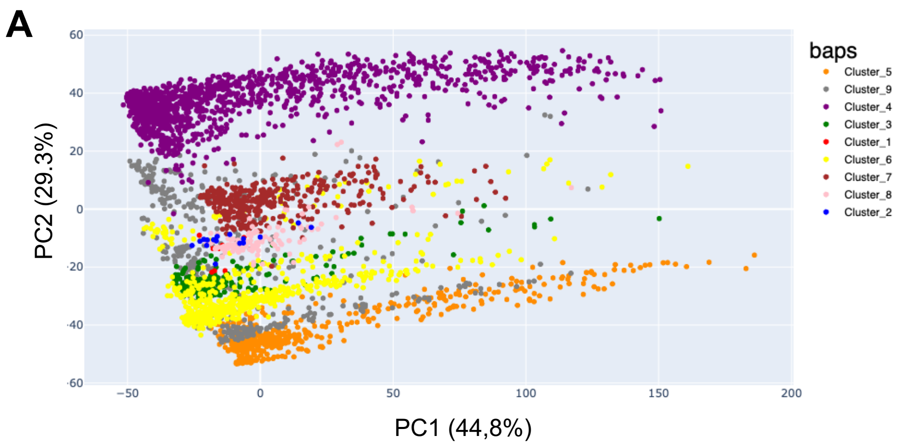

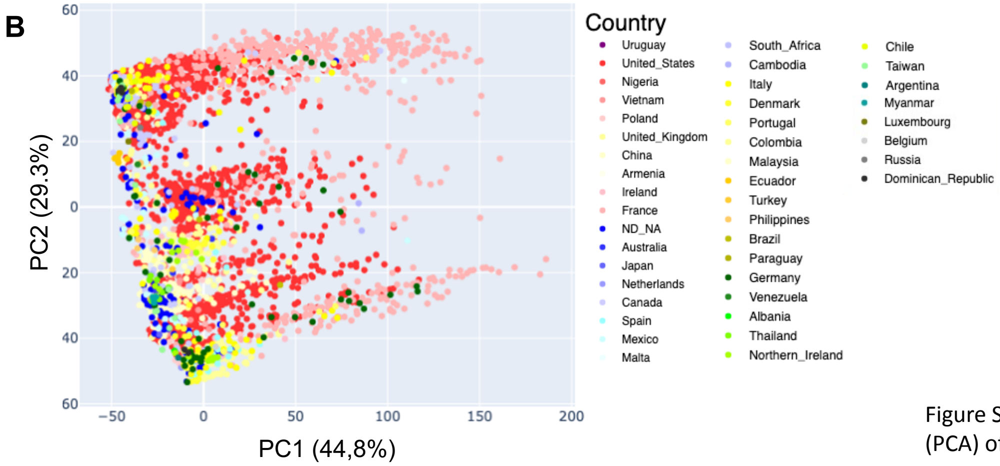

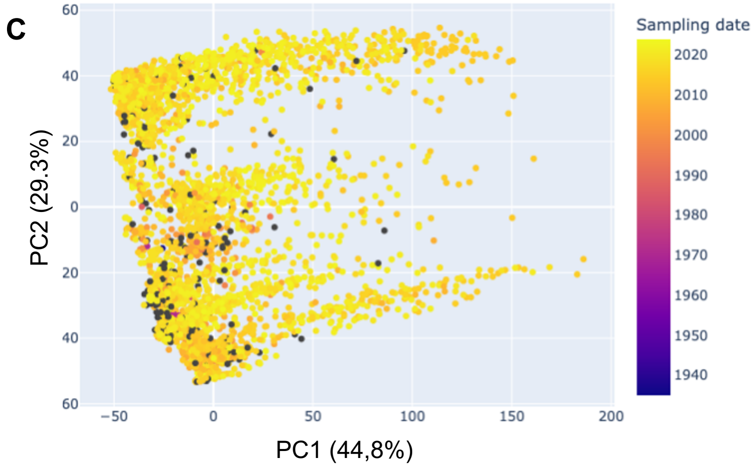

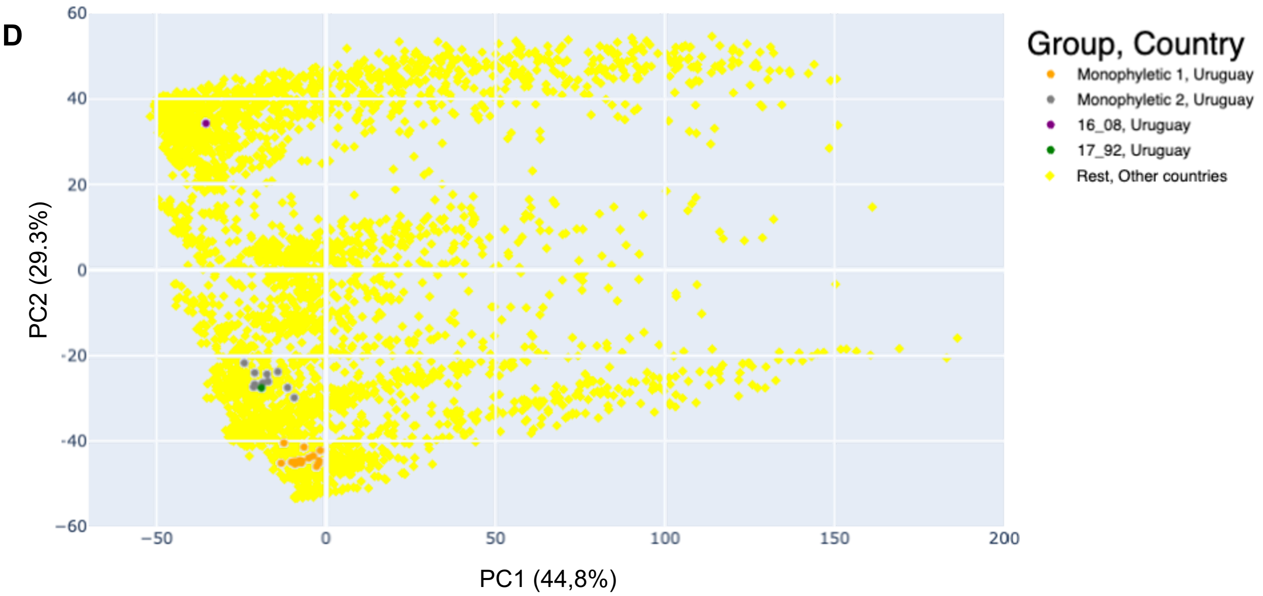

Figure S4. Principal component analysis (PCA) of the accessory genome. The PCA was conducted using a Jaccard distance matrix derived from the original absence/presence of genes as input data. Genes present in at least 15% and up to 95% of the analyzed genomes were included in the analysis. BAPS1, country of origin, and sampling date were superimposed onto the PCA using colors and are represented in panels A, B, and C, respectively. Panel D shows the distribution of Uruguayan isolates in the first two components of the PCA.
