# Supplementary material for: Genotypic and phenotypic analysis of Salmonella enterica serovar Derby, looking for clues explaining the impairment of egg isolates to cause human disease
Source: Front Microbiol. 2024 Jun 6;15:1357881. doi: 10.3389/fmicb.2024.1357881 (PMC11186997; doi:10.3389/fmicb.2024.1357881)
Supplement: Supplementary file 10 [file Image_5.pdf]

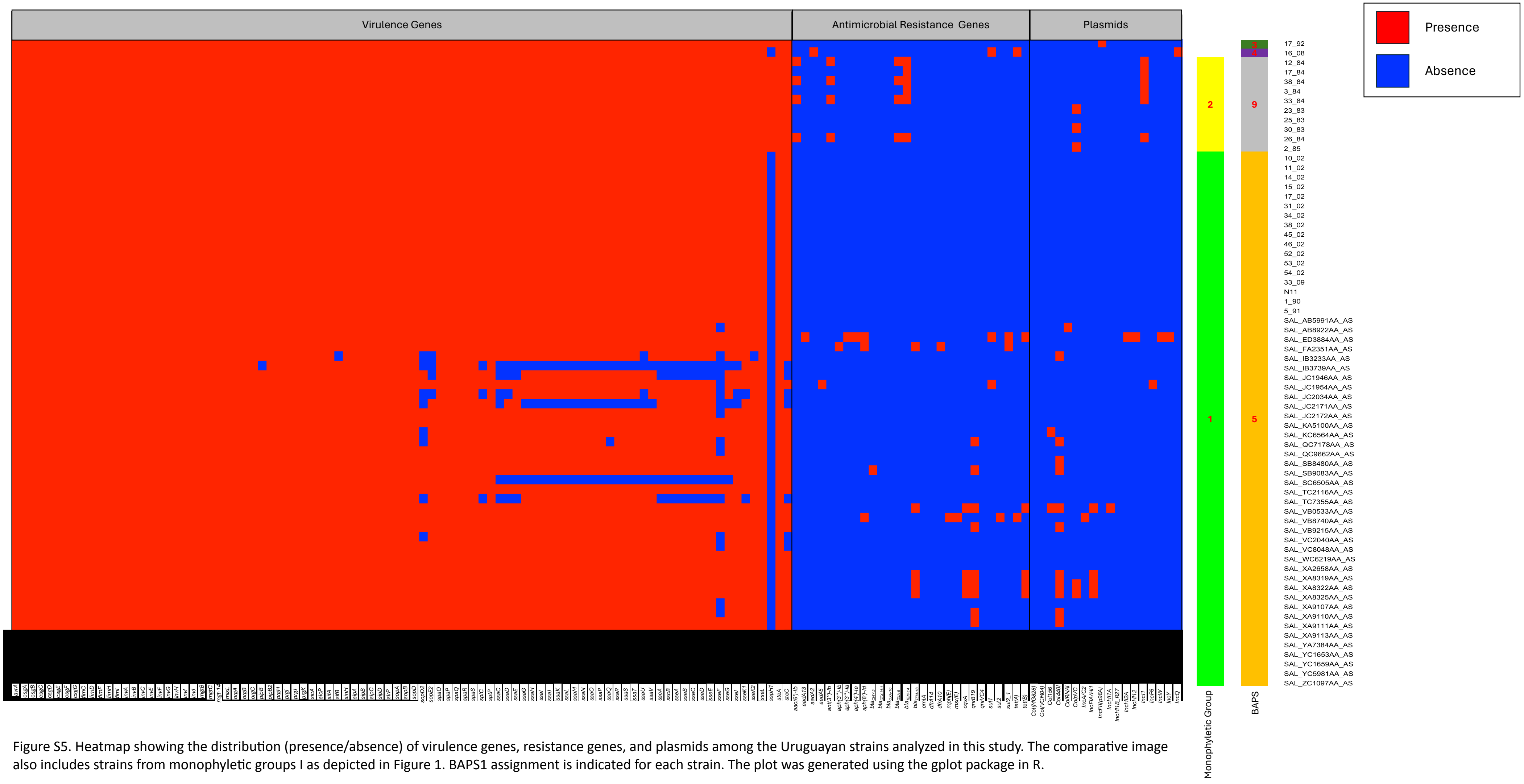

Figure S5. Heatmap showing the distribution (presence/absence) of virulence genes, resistance genes, and plasmids among the Uruguayan strains analyzed in this study. The comparative image also includes strains from monophyletic groups I as depicted in Figure 1. BAPS1 assignment is indicated for each strain. The plot was generated using the gplot package in R.
