# Supplementary material for: Genotypic and phenotypic analysis of Salmonella enterica serovar Derby, looking for clues explaining the impairment of egg isolates to cause human disease
Source: Front Microbiol. 2024 Jun 6;15:1357881. doi: 10.3389/fmicb.2024.1357881 (PMC11186997; doi:10.3389/fmicb.2024.1357881)
Supplement: Supplementary file 11 [file Image_6.PDF]

```
Monophyletic group II    1 : TTTACTCGCTTCGGTATGGATGGGATGGCAATGACCGGTATGCAGGTCAGCAGCCCATTATATCGTTTGCTGGCTCAGGTGACGCCAGAACAACGTGCGC : 100
Monophyletic group I     1 : TTTACTCGCTTCGGTATGGATGGGATGGCAATGACCGGTATGCAGGTCAGCAGCCCATTATATCGTTTGCTGGCTCAGGTGACGCCAGAACAACGTGCGC : 100
                                                             Fis site I                                Fis site II

Monophyletic group II  101 : CGGAGTAATCGTTTTCAGGTATATACCGGATGTTCATTGCTTTCAAAATTTTGCTATGTTGCCAGTATCCTTACGATGTATTTATTTTAAGGAAAAGCAT : 200
Monophyletic group I   101 : CGGAGTAATCGTTTTCAGGTATATACCGGATGTTCATTGCTTTCAAAATTTTGCTATGTTGCCAGTATCCTTACGATGTATTTATTTTAAGGAAAAGCAT : 200
                                  stop codon of          Fis site III                Fis site IV
                                  sseG

Monophyletic group II  201 : TATGGATATTGCACAATTAGTGGATATGCTCTCCCATATGGCGCACCAGGCAGGTCAGGCCATTAATGACAAAATGAATGGTAATGATTTGCTAAACCCA : 300
Monophyletic group I   201 : TATGGATATTGCACAATTAGTGGATATGCTCTCCCATATGGCGCACCAGGCAGGTCAGGCCATTAATGACAAAATGAATGGTAATGATTTGCTAAACCCA : 300
                             start codon of
                             ssaG

Monophyletic group II  301 : GA : 302
Monophyletic group I   301 : GA : 302
```

Figure S6. Nucleotide alignment of ssaG promoter region. The four Fis sites are indicated with a red letters. The best-matched sequence with the proposed 15-bp of highly degenerate Fis-binding site, Gnn(c/t)(A/g)(a/t)(a/t)(T/A)(t/a)(t/a)(T/c)(g/a)nnC (Lim et al., 2006), is indicated with green highlight. ATG, translation start codon of ssaG, and TAA, stop codon of sseG, are boxed with the yellow and grey highlight, respectively.
