# Supplementary material for: Genotypic and phenotypic analysis of Salmonella enterica serovar Derby, looking for clues explaining the impairment of egg isolates to cause human disease
Source: Front Microbiol. 2024 Jun 6;15:1357881. doi: 10.3389/fmicb.2024.1357881 (PMC11186997; doi:10.3389/fmicb.2024.1357881)
Supplement: Supplementary file 12 [file Image_7.PDF]

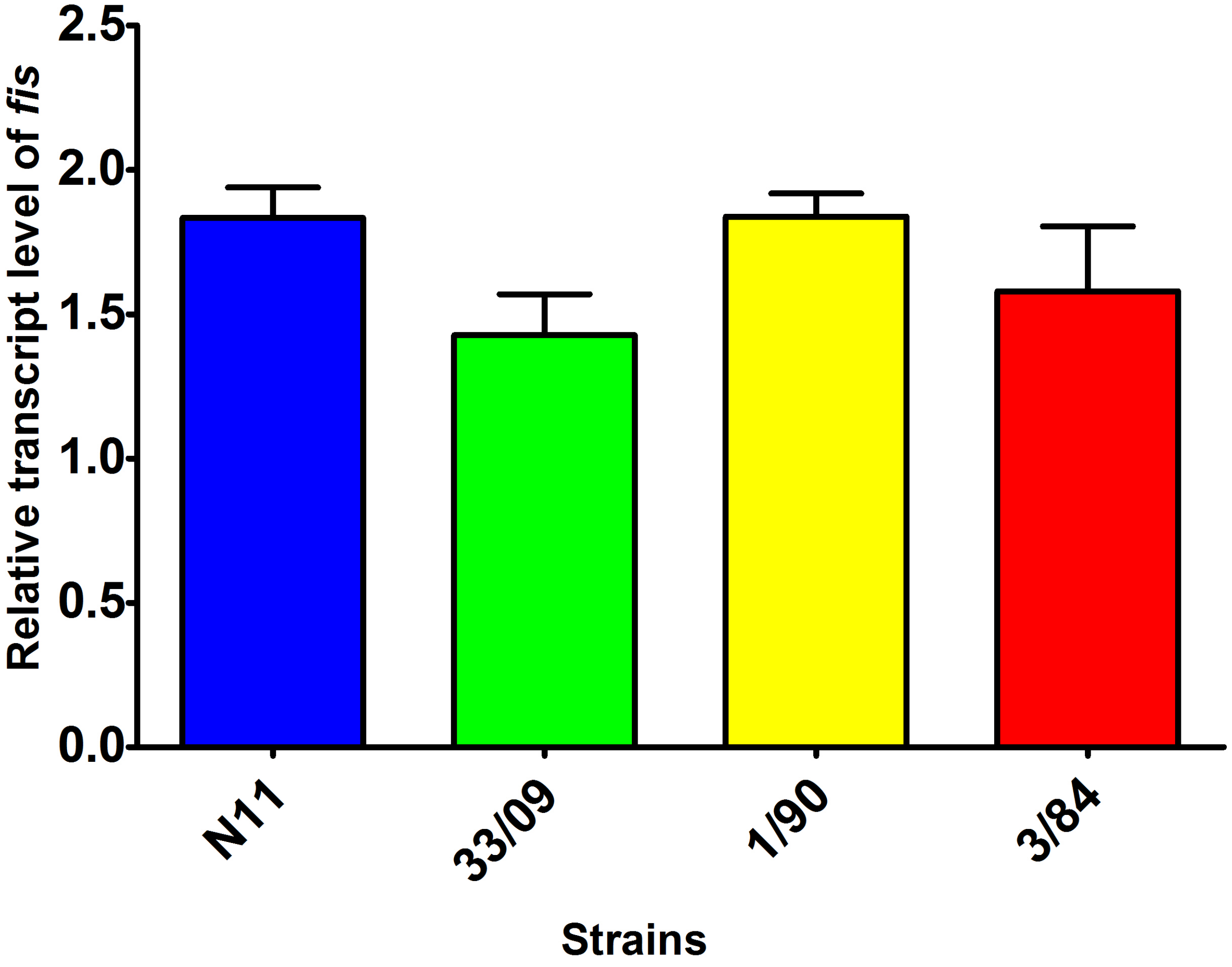

Figure S7. The relative expression levels of the fis gene were assessed under inductive conditions of ESP (OD600=2) in four representative strains of S. Derby. The analyzed isolates include three from monophyletic group I (N11 from eggs, 33/09, and 1/90 from humans) and one human isolate from monophyletic group II (3/84). No significant differences were observed between groups (Kruskal-Wallis and Dunn's post-analysis, $p<0.01$, $n=3$). The standard deviation is indicated by error bars.
