# Supplementary material for: Genotypic and phenotypic analysis of Salmonella enterica serovar Derby, looking for clues explaining the impairment of egg isolates to cause human disease
Source: Front Microbiol. 2024 Jun 6;15:1357881. doi: 10.3389/fmicb.2024.1357881 (PMC11186997; doi:10.3389/fmicb.2024.1357881)
Supplement: Supplementary file 13 [file Image_8.PDF]

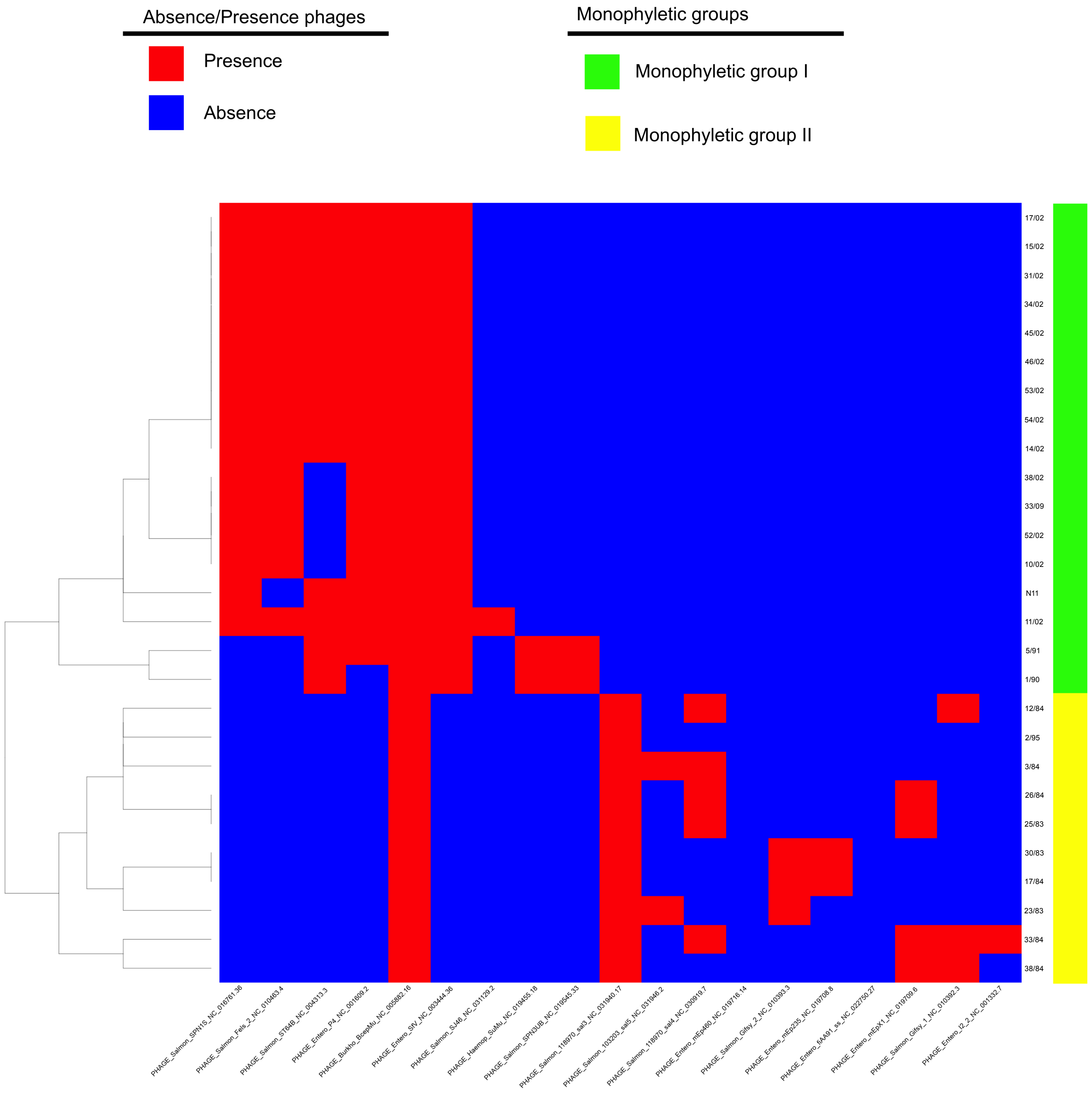

Figure S8. Comparison of putative prophages in Monophyletic groups I and II. The heat map represents the presence and absence of the 19 predicted putative prophages. Clustering was performed using the complete- linkage method along with Euclidean distance.
