# Supplementary material for: Genotypic and phenotypic analysis of Salmonella enterica serovar Derby, looking for clues explaining the impairment of egg isolates to cause human disease
Source: Front Microbiol. 2024 Jun 6;15:1357881. doi: 10.3389/fmicb.2024.1357881 (PMC11186997; doi:10.3389/fmicb.2024.1357881)
Supplement: Supplementary file 14 [file Image_9.PDF]

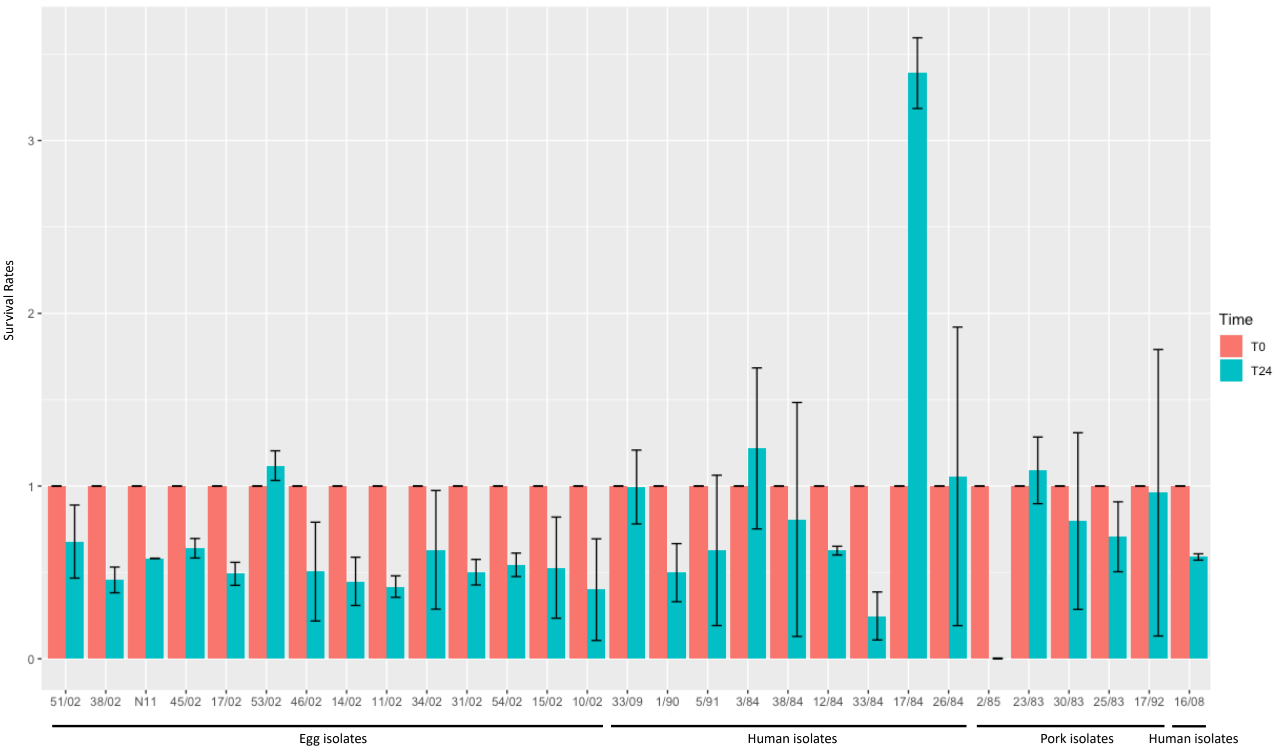

Figure S9. White egg survival assay of S. Derby isolates was conducted in four independent experiments. The survival rate was determined by calculating the ratio of bacterial cell counts at 24 hours (T24) to the initial bacterial cell counts (T0).
